# Angiotensin-Converting Enzyme 2 Activator Ameliorates Severe Pulmonary Hypertension in a Rat Model of Left Pneumonectomy Combined With VEGF Inhibition

**DOI:** 10.3389/fmed.2021.619133

**Published:** 2021-02-19

**Authors:** I-Chen Chen, Jao-Yu Lin, Yi-Ching Liu, Chee-Yin Chai, Jwu-Lai Yeh, Jong-Hau Hsu, Bin-Nan Wu, Zen-Kong Dai

**Affiliations:** ^1^Department of Pediatrics, Kaohsiung Medical University Hospital, Kaohsiung, Taiwan; ^2^Department of Pediatrics, School of Medicine, College of Medicine, Kaohsiung Medical University, Kaohsiung, Taiwan; ^3^College of Medicine, Graduate Institute of Medicine, Kaohsiung Medical University, Kaohsiung, Taiwan; ^4^Department of Surgery, Kaohsiung Medical University, Kaohsiung, Taiwan; ^5^Department of Pathology, School of Medicine, College of Medicine, Kaohsiung Medical University, Kaohsiung, Taiwan; ^6^Department of Pharmacology, School of Medicine, College of Medicine, Kaohsiung Medical University, Kaohsiung, Taiwan

**Keywords:** pulmonary arterial hypertension, left pneumonectomy, SU5416, ACE2 activator, diminazene aceturate, ACE2-Ang (1-7)-Mas axis

## Abstract

**Background:** Pulmonary arterial hypertension (PAH) is a life-threatening and deteriorating disease with no promising therapy available currently due to its diversity and complexity. An imbalance between vasoconstriction and vasodilation has been proposed as the mechanism of PAH. Angiotensin-converting enzyme 2 (ACE2), which catalyzes the hydrolysis of the vasoconstrictor angiotensin (Ang) II into the vasodilator Ang-(1-7), has been shown to be an important regulator of blood pressure and cardiovascular diseases. Herein we hypothesized diminazene aceturate (DIZE), an ACE2 activator, could ameliorate the development of PAH and pulmonary vascular remodeling.

**Methods:** A murine model of PAH was established using left pneumonectomy (PNx) on day 0 followed by injection of a single dose of the VEGF receptor-2 inhibitor SU5416 (25 mg/kg) subcutaneously on day 1. All hemodynamic and biochemical measurements were done at the end of the study on day 42. Animals were divided into 4 groups (*n* = 6–8/group): (1) sham-operated group, (2) vehicle-treatment group (SuPNx_42_), (3) early treatment group (SuPNx_42_/DIZE_1−42_) with DIZE at 15 mg/kg/day, subcutaneously from day 1 to day 42, and (4) late treatment group (SuPNx_42_/DIZE_29−42_) with DIZE from days 29–42.

**Results:** In both the early and late treatment groups, DIZE significantly attenuated the mean pulmonary artery pressure, pulmonary arteriolar remodeling, and right ventricle brain natriuretic peptide **(**BNP), as well as reversed the overexpression of ACE while up-regulating the expression of Ang-(1-7) when compared with the vehicle-treatment group. In addition, the early treatment group also significantly decreased plasma BNP and increased the expression of eNOS.

**Conclusions:** ACE2 activator has therapeutic potentials for preventing and attenuating the development of PAH in an animal model of left pneumonectomy combined with VEGF inhibition. Activation of ACE2 may thus be a useful therapeutic strategy for the treatment of human PAH.

## Introduction

Pulmonary hypertension (PH) is a progressive and presently incurable disorder, which complicates the majority of cardiovascular and respiratory diseases (1, 2). The etiology of PH is classified by the 6th World Symposium on Pulmonary Hypertension (WSPH) into the following five distinct groups: (1) Pulmonary arterial hypertension (PAH), (2) PH due to left heart disease, (3) PH due to lung disease and/or hypoxia, (4) PH due to pulmonary artery obstructions, and (5) PH with unclear and/or multifactorial mechanisms (3). All of these groups share a mean, resting, pulmonary arterial pressure (PAP) of >20 mmHg (3). The pathogenesis of PH is complexed and multifactorial; proliferative vasculopathy, pulmonary vascular remodeling, vascular constriction, and endothelial cell dysfunction have been proven in several previous studies (1).

The Renin-angiotensin system (RAS) has been implicated in playing a causative role in PH (4–13). There are two opposing arms in RAS: the presser arm, composed of angiotensin converting enzyme (ACE), angiotensin II (Ang II) produced from Ang I by ACE, and the Ang II type 1 (AT1) receptor as the main protein mediating the biological actions of Ang II; and the vasodilator arm, consisting angiotensin converting enzyme 2 (ACE2), Ang-(1-7) generated through hydrolysis of Ang II by ACE2, and the Mas receptor as the protein conveying the vasodilatory, antiproliferative, antifibrotic, and antithrombotic effects of Ang-(1-7) (4–16). Therefore, an increase in the levels of Ang-(1-7) should have beneficial effects for PAH. However, targeting the ACE2/Ang (1-7)/Mas receptor pathway in PAH is still under scrutiny.

ACE2 has exhibited its modulating ability in the balance between vasoconstriction and vasodilation in many experiments (5, 10, 11, 13). Recently, diminazene aceturate (DIZE), a Food and Drug Administration (FDA) approved anti-trypanosomal drug, has been demonstrated to exert off-target effect on activating ACE2 (17, 18). This effect of DIZE has been confirmed through the analysis of cleavage of the vasoconstrictor peptide Ang II, the most physiologically relevant natural substrate for ACE2 (17).

It has also been shown that the SU5416 hypoxia model developed pulmonary arterial changes resembling plexiform-like lesions, which are characteristic features of human PAH, by increasing apoptosis of endothelial cells followed by converting them into apoptosis resistant and phenotypically altered endothelial cells (19). Recently, a newly developed rodent PAH model that combined left pneumonectomy with SU5416 was reported (20). Unlike the traditional SU5416 hypoxia model in which a partial reversal of PAH is seen upon returning to normoxia, this new animal model does not reverse hypoxia-induced vasoconstriction and hemoconcentration under similar conditions. Rather, the right ventricular systolic pressure increased gradually over time, a feature that may favor the assessment of drug effects in preclinical trials (20).

On the basis of the benefits demonstrated by DIZE in other PH models (18, 21–23), we aimed to evaluate its effects on a rat model of PAH induced by left pneumonectomy combined with SU5416. We reasoned that in this model there could be an up-regulated expression of ACE, leading to the imbalance between vasoconstriction and vasodilation. Treatment with DIZE would increase the levels of ACE2, and hence, the vasodilatory peptide Ang-(1-7) to prevent and ameliorate the development of PAH.

## Methods

### Animal Model of PAH

All protocols had been approved by the animal research committee of Kaohsiung Medical University (IACUC approval No. 104226). Male Wistar rats weighing ~220 g were purchased from National Animal Center, Taiwan. after arriving at the Kaohsiung Medical University vivarium, the rats were acclimated for at least 1 week before being used in the experiment. They were housed in a room on a 12-h light/dark cycle under controlled temperature of 22.1°C and relative humidity of 55%, and they were provided with normal chow and water *ad libitum*. On the day of experiment, animals were anesthetized with a mixture of sodium pentobarbitone (20 mg/kg, i.p.) and ketamine (40 mg/kg, i.m.) under orotracheally ventilated rodent respirators (Harvard, South Natick, MA) and lateral thoracotomy in the third intercostal space was performed. Left lung of rats was resected and closed by clip ligation (Ligaclip multiple clip applier, medium size, Ethicon Endo-Surgery), and the animals were suture to prevent pneumothorax. The chest retractor was then removed after 15 min, and the thymus was moved back to its physiological position. The chest cavity and skin were closed. The entire procedure took <30 min for each rat. After operation, rats were extubated and received an additional injection of sterile saline (10 ml). On the following day, SU5416 (25 mg/kg) was injected subcutaneously in surviving rats. Sham-operated animals underwent identical operation without pneumonectomy (PNx) and received sterile saline (20 mg/kg) subcutaneously, instead. The day of SU5416 injection was designated as day 0. All animals were individually housed in a 12-h dark/light cycle-controlled room and fed a regular rat diet. The procedure was also shown in Supplementary Figure 1.

### Study Design

**Figure 1:**
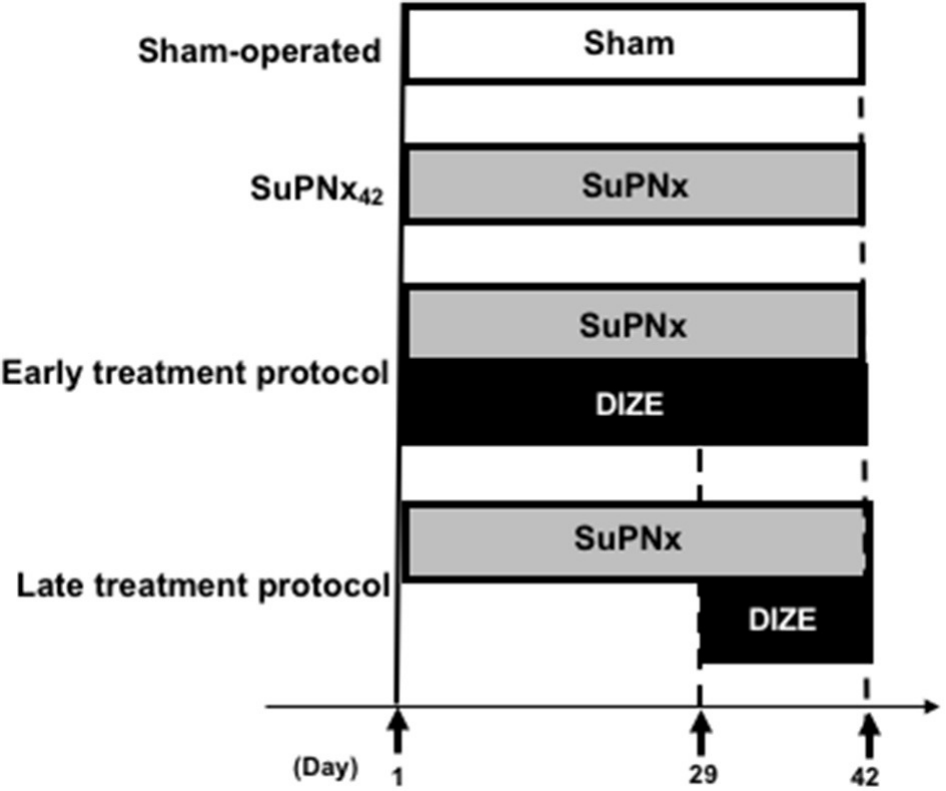
The design of animal study. In the early treatment protocol, there were sham-operated rats, SU5416 combined with left pneumonectomy rats (SuPNx_42_), and SuPNx_42_ rats given with 15 mg DIZE/kg/day from day 1 to day 42 (SuPNx_42_/DIZE_1−42_). In the late treatment protocol, there were sham-operated rats (sham42), SuPNx_42_, and a SuPNx_42_ rats given with 15 mg DIZE/kg/day from day 29 to day 42 (SuPNx_42_/DIZE_29−42_). SuPNx, Su5416 with left pneumonectomy; DIZE, diminazene aceturate.

The study design was shown in Figure 1. All rats were randomized (*n* = 6–8/group) to undergo the sham operation (group 1) or SU5416 injection with left pneumonectomy (SuPNx). The SuPNx rats were further randomized for subcutaneous treatment with saline (SuPNx_42_) (group 2) or DIZE (Santa Cruz Biotechnology, CA, USA) at a dose of 15 mg/kg/day from day 1 to day 42 (SuPNx_42_/DIZE_1−42_) in the early treatment protocol (group 3) or from day 29 to day 42 (SuPNx_42_/DIZE_29−42_) in the late treatment protocol (group 4). On day 42, all rats were ventilated and sacrificed after checking hemodynamic data. The lungs and heart from each rat were rapidly perfused with sterile saline under a pressure of 100-cm H_2_O prior to removal. One-half of the lung tissue was homogenized for protein extraction, and the other half was kept in 10% formaldehyde and froze in 4°C for histopathologic analysis.

### Hemodynamics Measurements

The method was described in our previous published study (24, 25). Briefly, a PE-50 catheter (Beckton-Dickinson, Sparks, MD, USA) was inserted into the femoral artery using a cut-down procedure for recording pressure. Thereafter, a left parasternal thoracotomy was performed. Another catheter was inserted closely to the main pulmonary artery *via* the right ventricular outflow tract. Pulmonary and femoral arterial pressures were recorded simultaneously.

### Plasma and Right Ventricle Brain Natriuretic Peptide (BNP)

Plasma samples (1.0 mL) and homogenous right ventricle (RV) were expressed in v/v for TFA and homogenate in w/v and then centrifuged at 2,000 × g for 15 min at 4°C. Enzyme-immunoassay kits (Phoenix Pharmaceuticals, Burlingame, CA, USA) were used to measure plasma and RV BNP contents according to the manufactural protocol.

### Histopathologic and Immunohistochemical Analysis

Lung tissues which were immersed in formalin for at least 24 h were analyzed for histopathology. Hematoxylin-Eosin (H-E) staining was used for analysis of the pulmonary arterioles in terms of medial wall thickness under a microscope at a magnification of 400X. The methods were described in our previous published study (24, 25) and the medial wall thickness of each arteriole was expressed as follows:

Percent wall thickness = (medial thickness × 2 /external diameter) × 100.

Verhoeff-van Gieson staining was performed for evaluating the severity of neointimal formation using a modified scoring system published before (26–28). The degree of vascular remodeling was determined by measuring 30 transversally cut vessels (outer diameter: 50–100 μm) from each rat and expressing the thickness of the combined intimal and medial layers as a percentage of total vessel diameter. The percentage of vascular occlusion of these 30 vessels was categorized as Grade 0: no evidence of luminal obstruction; Grade 1: the presence of partial (<50%) luminal occlusion; and Grade 2: the present of luminal occlusion >50%. An average Grade score for 30 vessels was calculated for each animal. Afterwards, a quantitative analysis of luminal obstruction in 30 consecutive small pulmonary arteries from all rats in each group was performed.

In addition, double immunohistochemical staining was performed to present the smooth muscle actin (SMA) by using formalin-fixed, paraffin-embedded tissue. Experiments were performed by double staining polymer detection systems (TADS03, BioTnA, Taiwan) using rabbit polyclonal anti-SMA (1:100, Cell signaling, MA, USA) and anti-PCNA (Proliferating Cell Nuclear Antigen) (1:100, Cell signaling, MA, USA) antibodies.

### Western Blot Analysis of Pulmonary eNOS, ACE, ACE2, and MAS

Lung tissues(100 mg) were homogenized in 1 ml of RIPA buffer [1% Triton X-100, 15 mM HEPES-NaOH (pH 7.5), 0.15 mM NaCl, 1% sodium deoxycholate, 0.1% SDS, 1 mM sodium orthovanadate, 10 mM EDTA, and 0.5% protease inhibitor cocktail (Sigma, St. Louis, MO, USA)] and centrifuged at 15,000 × g for 20 min at 4°C. Proteins in the supernatant (100 μg) were subjected to SDS-PAGE in 10% polyacrylamide gels and were transferred onto PVDF membranes (Pall, Port Washington, NY, USA). Each membrane was blocked with 5% non-fat dry milk in Tris-buffered saline (TBS), probed with anti-actin (1:10,000) (Upstate Biotechnology, Lake Placid, NY, USA), anti-eNOS (1:1,000, Millipore, Milford, MA, USA) anti-ACE, anti-ACE2 (1:500 dilution; Millipore, Billerica, MA, USA), or anti-MAS antibodies (1:1,000 dilution; Millipore), and then incubated with horseradish peroxidase-conjugated secondary antibody (Leadgene Biomedical, TW). Signals were detected using the Western Lighting® chemiluminescent kit (Millipore) according to the manufacturer's specifications.

### Ang II and Ang-(1-7) Measurements

RV and lung supernatants were acidified with 0.6% trifluoroacetic acid (TFA) to obtain a 10% (w/v) homogenate. The samples were centrifuged at 2,000 × g for 15 min at 4°C, and dried under a steam of nitrogen at 60°C. Enzyme-immunoassay kits (Phoenix Pharmaceuticals, CA, USA) were used to measure the Ang II and Ang-(1-7) contents in the RV and lung.

### ACE2 Activity Assay

The method was modified as previously described (29). Lysates were prepared by homogenizing ~100 mg tissue sections in ACE2 lysis buffer (BioVision Inc.). They were kept on ice for 10 min followed by gentle vortexing followed by another 5 min on ice. The tissue lysates then were centrifuged at 16,000 g at 4°C for 10 min and the pellets discarded. The protein concentrations of tissue lysates were measured using a bicinchoninic acid method (#K818, BioVision Inc.). Activity of ACE2 in tissue lysates was measured according to the manufacturer's instructions.

### Statistical Analysis

The results obtained from ELISA and Western blots were analyzed by densitometry and expressed as means ± standard error of the mean. All data from the four groups were analyzed using one-way analysis of variance (ANOVA) followed by *post-hoc* Tukey's test (*n* = 6–8 rats/group). A *P* < 0.05 was considered statistically significant.

## Results

### DIZE Reduced RV Systolic Pressure and RV and Plasma BNP

**Figure 2:**
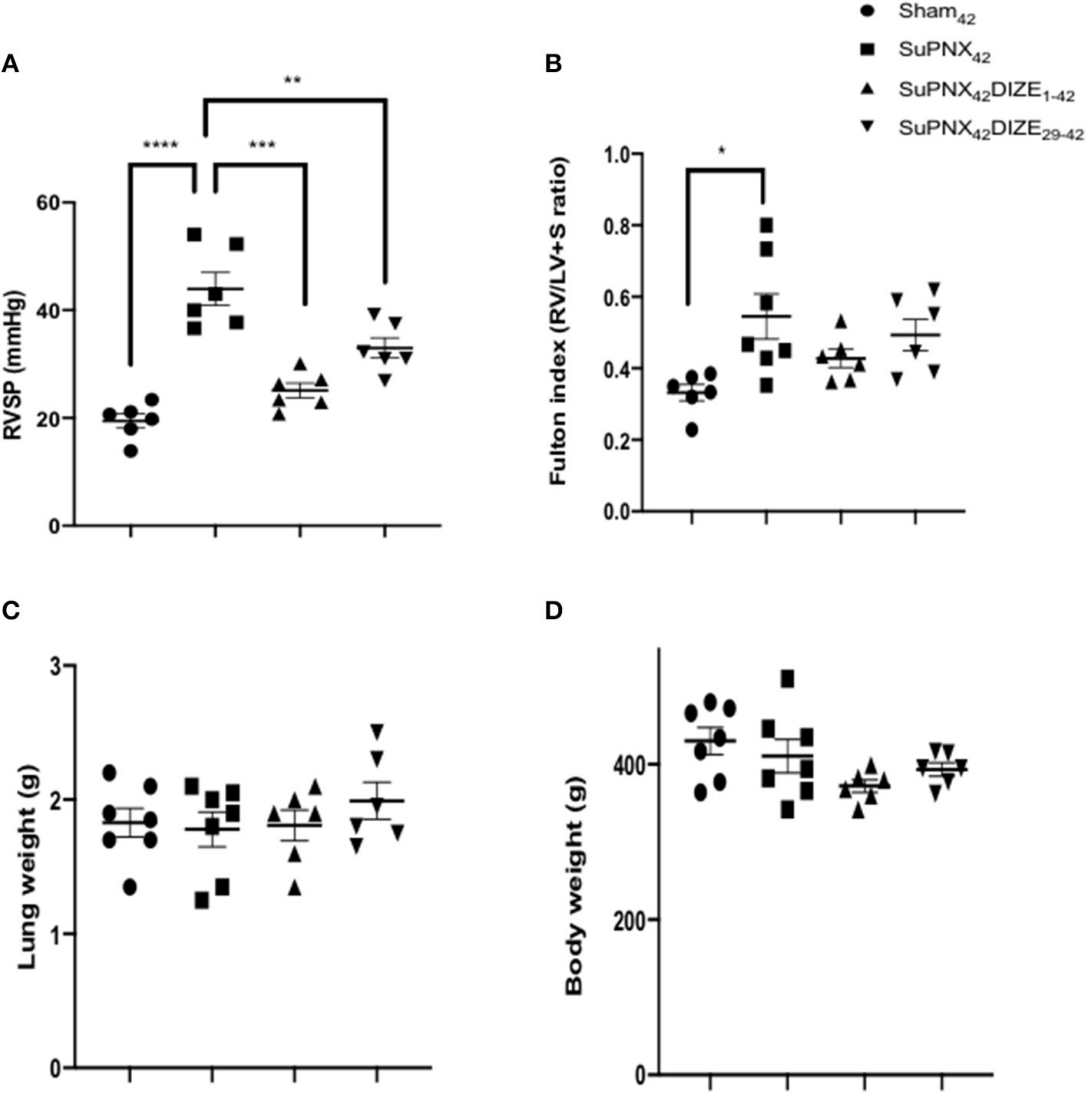
Comparison of **(A)** right ventricle systolic pressure (RVSP), **(B)** Fulton index, the weight ratio of right ventricle to left ventricle plus septum (RV/LV+S), **(C)** the lung weight and **(D)** the body weight among the sham_42_, SuPNx_42_, SuPNx_42_/DIZE_1−42_ and SuPNx_42_/DIZE_29−42_ groups. RVSP levels were significantly higher in the SuPNx_42_ group (45.17 ± 4.62 mmHg) than in sham-operated rats (18.37 ± 1.36 mmHg, *p* < 0.001). Both DIZE treatment groups (SuPNx_42_/DIZE_1−42_ and SuPNx_42_/DIZE_29−42_) showed significant decreases in RVSP when compared with that of the SuPNx_42_ rats (early, 25.10 ± 1.38 mmHg, *p* < 0.01; late 31.23 ± 4.06 mmHg, *p* < 0.05). The Fulton index was significantly increased in the SuPNx_42_ group (0.55 ± 0.06) than in sham-operated group (0.35 ± 0.04, *p* < 0.05), but no significant changes were observed in the SuPNx_42_/DIZE_1−42_ and SuPNx_42_/DIZE_29−42_ groups when compared with the SuPNx_42_ group. There was no significant change in either the lung or body weight in all groups. Values represent the mean ± SEM. ^*^P < 0.05, ^**^P < 0.01, ^***^P < 0.001, ^****^P < 0.0001 (*n* = 6–7).

DIZE did not alter the mean systolic arterial pressure (mSAP) in both early (SuPNx_42_/DIZE_1−42_) and late DIZE treatment (SuPNx_42_/DIZe_29−42_) groups (Supplementary Figure 2). Significant increases in the right ventricular systolic pressure (RVSP) were observed in the SuPNx_42_ groups when compared with the sham-operated rats (Figure 2A). Both early (SuPNx_42_/DIZE_1−42_) and late DIZE treatment (SuPNx_42_/DIZe_29−42_) groups decreased the RVSP when compared with the vehicle treatment group (Figure 2A). The Fulton index, weight ratio of right ventricle to left ventricle plus septum (RV/LV+S), in the SuPNx42 rats was significantly increased than the sham-operated rats, which indicated the right ventricle was hypertrophied in the SuPNx42 animals (Figure 2B). However, the RV/LV+S ratio in either the early or late DIZE treatment group was not different when compared to SuPNx_42_ group (Figure 2B). There were no significant changes among all 4 groups in the lung or body weight (Figures 2C,D).

**Figure 3:**
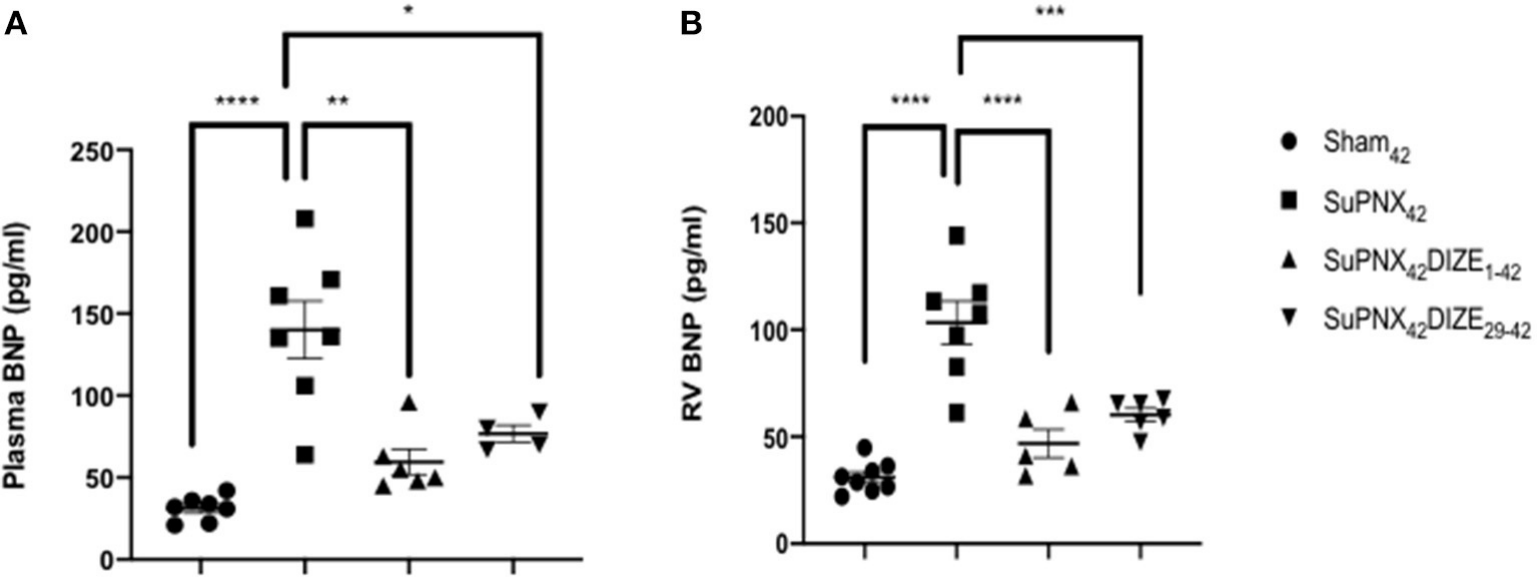
**(A)** The plasma brain natriuretic peptide (BNP) and **(B)** RV BNP levels. The plasma BNP level was significantly higher in the SuPNx_42_ rats than in sham-operated rats (130.2 ± 19.76 vs. 59.50 ± 7.74 pg/ml; *P* < 0.001). Treatment with DIZE for 42 days significantly lowered the plasma BNP levels when compared with that of the vehicle-treated group (31.14 ± 2.83 vs. 130.2 ± 19.76 pg/ml; *P* < 0.05). There was a trend that the SuPNx_42_/DIZE_29−42_ rats had a decreased plasma BNP level when compared with the SuPNx_42_ rats, but the difference was not statistically significant. The RV BNP was significantly higher in SuPNx_42_ rats than that of the sham_42_ rats (103.4 ± 10.01 vs. 31.05 ± 2.57 pg/ml; *P* < 0.0001). Both DIZE treatment groups had significantly lowered RV BNP than in the SuPNx_42_ rats (early: 46.82 ± 6.62 pg/ml vs. 103.4 ± 10.01 pg/ml, *P* < 0.0001; late: 61.83 ± 5.10 pg/ml vs. 103.4 ± 10.01 pg/ml, *P* < 0.001). Values represent the mean ± SEM. ^*^P < 0.05, ^**^P < 0.01, ^***^P < 0.001, ^****^P < 0.0001 (*n* = 6–8).

Plasma BNP, an index of heart failure, was elevated significantly in the SuPNx_42_ group compared with the sham-operated rats. In the early treatment group (SuPNx_42_/DIZE_1−42_), DIZE resulted in significant decreases in plasma BNP compared to that of vehicle-treated SuPNx_42_ rats, but no significant changes were seen in the late treatment group (SuPNx_42_/DIZE_29−42_) (Figure 3A).

Similar to the plasma BNP, RV BNP was also elevated significantly in SuPNx_42_ group compared with the sham-operated rats. Both the early treatment group (SuPNx_42_/DIZE_1−42_) and the late treatment group (SuPNx_42_/DIZE_29−42_) displayed significant decreases in the levels of RV BNP compared to that found in the vehicle-treated SuPNx_42_ rats (Figure 3B).

Ang II, which induces vasoconstriction, has a trend of higher activity in SuPNX_42_ rats than in matched sham_42_ rats (*p* = 0.0528). However, neither the early nor the late DIZE treatment protocol altered the level of Ang II in the lung (Supplementary Figure 3).

### DIZE Attenuated Pulmonary Vascular Remodeling and Smooth Muscle Neointimal Proliferation

**Figure 4:**
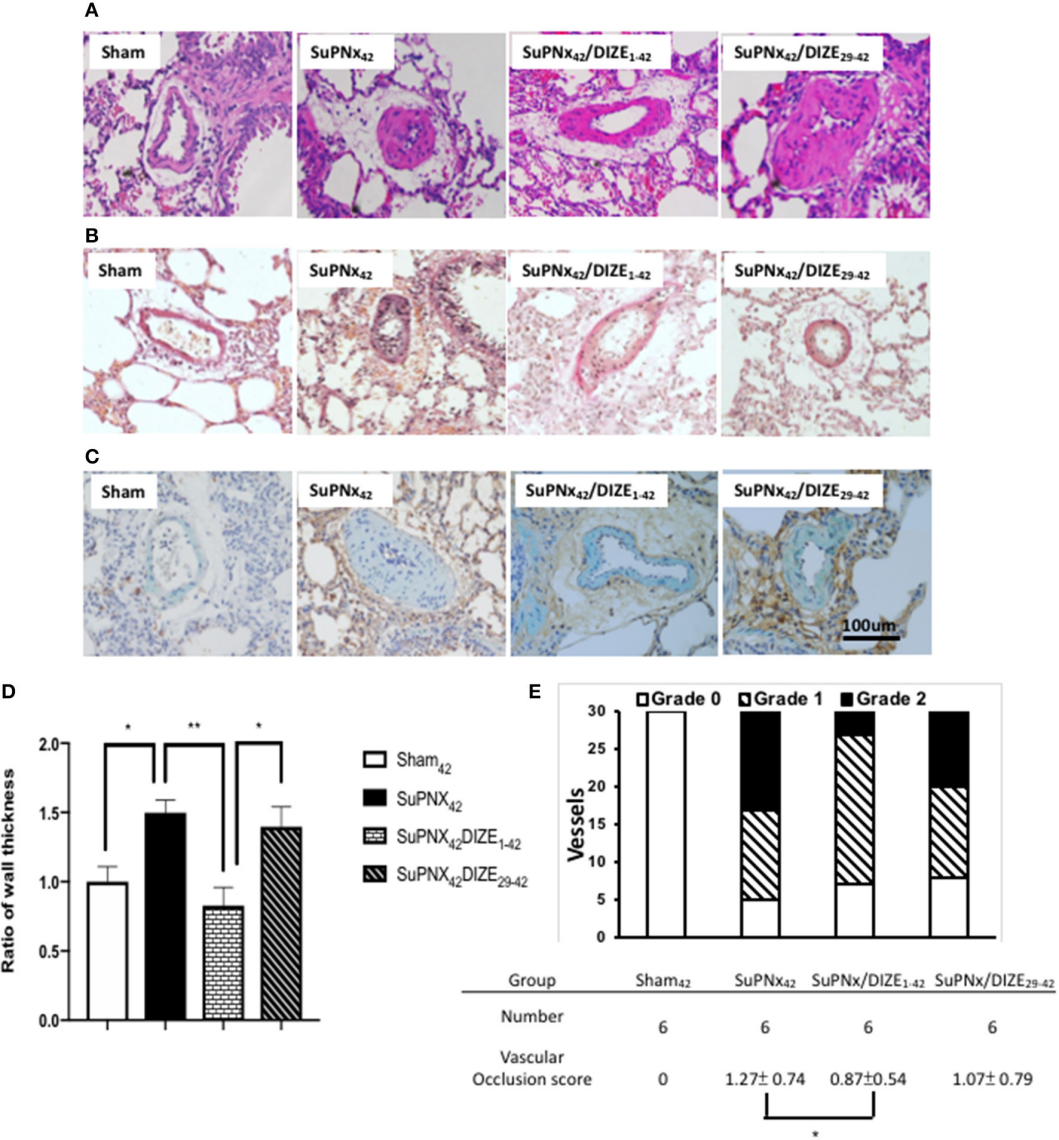
The representative results of Hematoxylin-eosin stating of lung tissue **(A)**, Verhoeff-van Gieson staining **(B)**, and double immunohistochemical staining of smooth muscle actin (green) and PCNA (brown) **(C)**. **(D)** The ratio of medial wall thickness of pulmonary arterioles. The value for the sham_42_ group was set at 1.0. The muscular layer of pulmonary arterioles (50–100 μm in diameter) was significantly thicker in the SuPNx_42_ rats than in sham_42_, SuPNx_42_/DIZE_1−42_, but not SuPNx_42_/DIZE_29−42_ rats (*n* = 6). **(E)** the vascular occlusion score (VOS). The VOS of early treatment group (SuPNx_42_/DIZE_1−42_) was decreased compared to SuPNx_42_ (1.27 ± 0.74 vs. 1.07 ± 0.56, *n* = 6) (magnification, 200x. Values represent the mean ± SEM. ^*^P < 0.05, ^**^P < 0.01.

Using H-E staining, the medial wall thickness of pulmonary arterioles (50–100 μm in diameter) was found to increase significantly in the SuPNX_42_ rats compared with that in sham-operated rats (Figures 4A,D). Early DIZE treatment (SuPNx_42_/DIZE_1−42_) significantly attenuated the medial wall thickness of pulmonary arterioles, when compared with the vehicle-treated rats (SuPNx_4_) (Figures 4A,D). However, no significant changes in medial wall thickness were observed in the SuPNx_42_/DIZE_29−42_ group when compared with that of the SuPNx_42_ rats.

In addition, Verhoeff-van Gieson staining was carried out for evaluating the severity of neointimal formation (Figure 4B). Double immunohistochemical staining of smooth muscle actin was shown in Figure 4C. The distribution of vascular lesions and an average vascular occlusion score (VOS) are presented in Figure 4E. The SuPNx_42_ rats developed severe pulmonary vascular remodeling with a VOS of 1.27 ± 0.74. In the early treatment group with DIZE, the VOS was significant decreased (0.87 ± 0.54) compared to the SuPNx_42_ rats (*p* < 0.05). There was also a trend of decrease in the late treatment group with a VOS of 1.07 ± 0.79 when compared to the SuPNx_42_ rats_._

### DIZE Regulated the Expressions of eNOS, ACE, and ACE2/MAS Axis in SuPNx Rats

**Figure 5:**
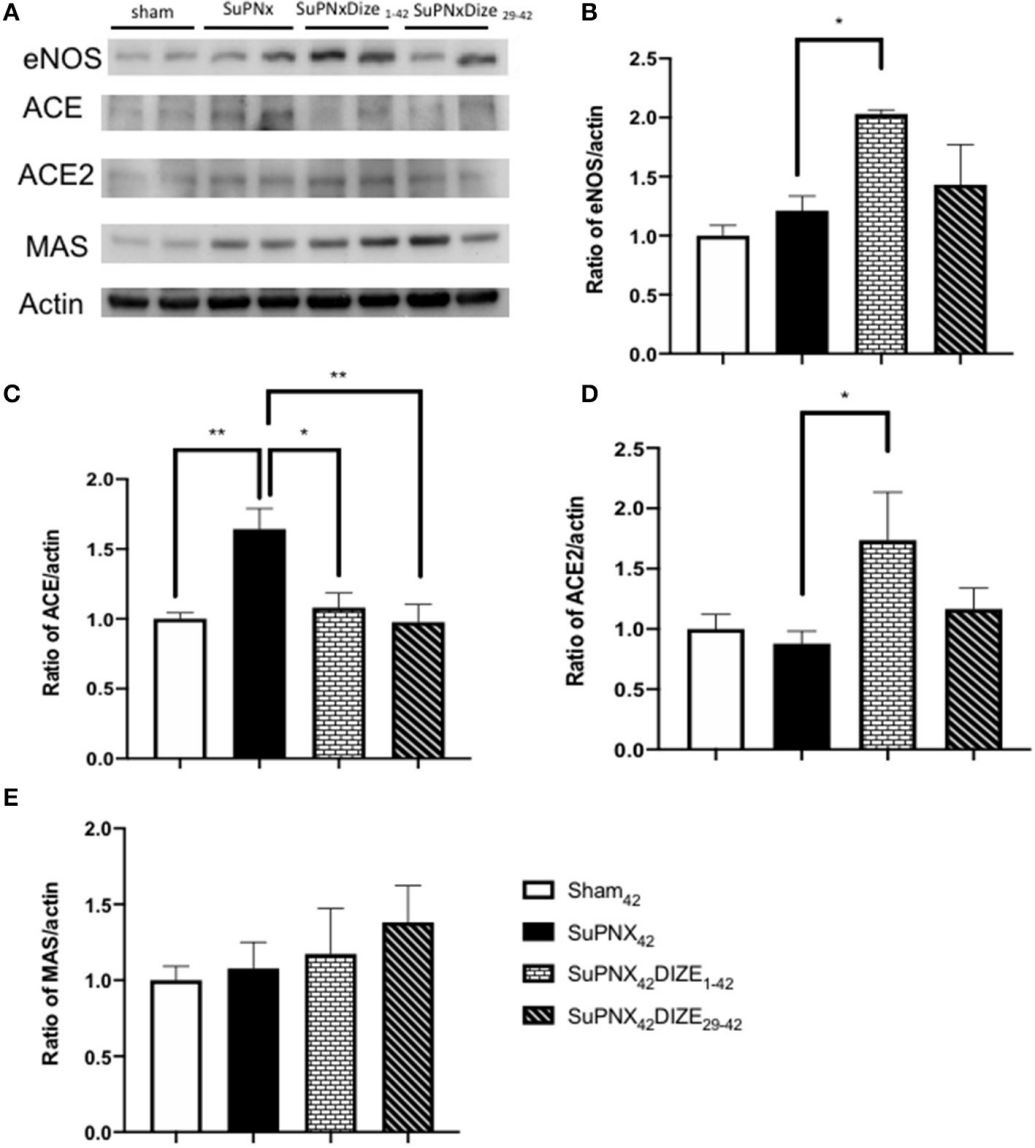
**(A)** A representative Western blot of eNOS, ACE, ACE2, and the MAS receptor in sham_42_, SuPNx_42_, SuPNx_42_/DIZE_1−42_, and SuPNx_42_/DIZE_29−42_ rats. Representative results from one of three separate Western blotting experiments are shown. **(B)** The normalized eNOS/actin ratio revealed the expression of eNOS was higher in the SuPNx_42_/DIZE_1−42_ rats than in SuPNx_42_ rats. **(C)** The normalized ratio of ACE/actin showed that the expression of ACE was higher in SuPNx_42_ rats than in sham_42_ rats. DIZE administration decreased ACE expression in the early treatment (SuPNx_42_/DIZE_1−42_) and the late treatment (SuPNX_42_/DIZE_29−42_) groups. **(D)** The normalized ratio of ACE2/actin revealed that an increase in the early treatment group (SuPNX_42_/DIZE_1−42_) compared to the SuPNx_42_ rats. No significant differences were found among the sham_42_, SuPNx_42_, and SuPNX_42_/DIZE_29−42_ groups. **(E)** The normalized MAS/actin ratios were not changed in the all groups. The value for the sham_42_ group was set at 1.0 in **(B–E)**. Values represent the mean ± SEM ^*^P < 0.05, ^**^P < 0.01 (*n* = 6–7).

Figure 5A shows a representative pulmonary expression of eNOS, ACE, ACE2, MAS by Western blot analysis. SuPNX_42_ has the trend to upregulated pulmonary eNOS expression when compared with the sham-operated rats. Pulmonary expression of eNOS was significantly elevated in the early treatment protocol when compared with the SuPNx_42_ groups, but this was not seen in the late treatment protocol (Figure 5B).

Compared to the sham-operated rats, the ratio of pulmonary expression of ACE was elevated in the SuPNx_42_ group. Significant decreases in ACE protein expression in both early and late DIZE treatment groups were noted when compared with the vehicle-treated SuPNx_42_ rats (Figure 5C).

Pulmonary expression of ACE2 was increased in the early treatment group (SuPNX_42_/DIZE_1−42_) compared to the SuPNx_42_ rats. No significant differences were found among the sham_42_, SuPNx_42_, and SuPNX_42_/DIZE_29−42_ groups (Figure 5D). The ACE2 activity of lung was also shown in the Supplementary Figure 4.

The Mas is a G protein-coupled receptor with its vasoprotective effect in RAS system (16). In western blot analysis, there were no significant changes in Mas expression among all four groups (Figure 5E).

### DIZE Upregulated RV and Pulmonary Ang-(1-7) Levels

**Figure 6:**
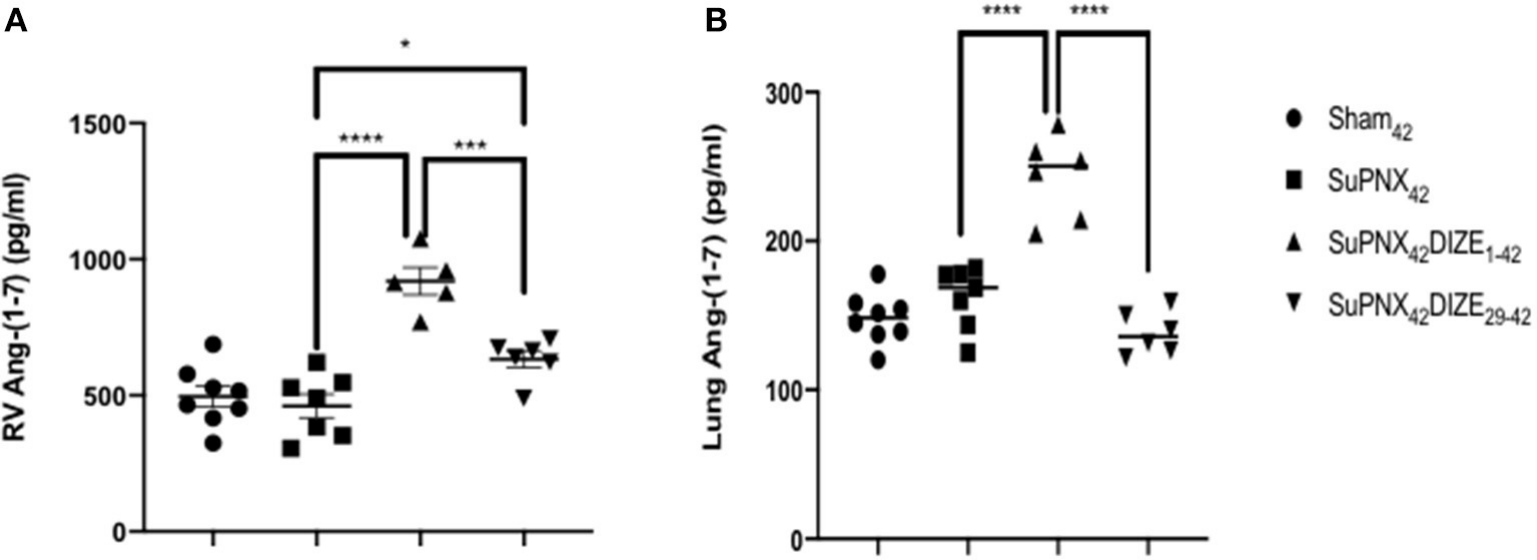
**(A)** The levels of RV Ang-(1-7). The level of RV Ang-(1-7) was significantly higher in the SuPNx_42_ /DIZE_1−42_ and SuPNx_42_ /DIZE_29−42_ rats than in SuPNx_42_ rats. **(B)** The levels of pulmonary Ang-(1-7). The expression of pulmonary Ang-(1-7) was significantly higher in SuPNx_42_ /DIZE_1−42_ rats than in SuPNx_42_ rats. Values represent the mean ± SEM. ^*^P < 0.05, ^***^P < 0.001, ^****^P < 0.0001 (*n* = 6–7).

The RV and pulmonary tissue Ang-(1-7) levels were not changed in the SuPNx_42_ group when compared to the sham-operated rats (Figures 6A,B). However, there was a significant increase of RV Ang-(1-7) in both the early and late DIZE treatment groups when compared with the vehicle-treated SuPNx_42_ rats (Figures 6A,B). In addition, early DIZE treatment also increased the levels of pulmonary Ang-(1-7) when compared with the SuPNx_42_ group (Figure 6B). No significant differences were found among the sham_42_, SuPNx_42_, and SuPNx_42_/DIZE_29−42_ groups (Figure 6B).

## Discussion

SU5416 was the first VEGF receptor 2 inhibitor to enter clinical development for cancer therapy (30). It has been shown that inhibiting the VEGF- and endothelial-dependent proliferation will result in structural changes known as plexiform lesions (19, 30–33). Allowing for the selection of an apoptosis resistant, proliferating endothelial cell phenotype by the combination of blockade of VEGF receptor 2 and hypoxia, severe PAH will develop (30). In this study, we reasoned that a pulmonary insult such as pneumonectomy followed by injection with SU5416 will yield similar results. In fact, our SuPNx model was shown to cause severe angio-obliterative PAH associated with increased cell proliferation and proapoptotic signaling, resulting in neointimal and medial remodeling (20). In addition, unlike the hypoxia model where partial reversibility of pulmonary hypertension is seen after returning to normoxia, the SuPNx model is independent of hypoxic vasoconstriction and hemoconcentration (20).

Accumulating evidence has shown that in human and animal models, RAS activity is increased in PH. However, therapies targeting RAS-signaling pathway, such ACE inhibitors or AT1 receptor blockers, were susceptible to develop hypotension due to right ventricular dysfunction in patients with PH (11). In contrast, DIZE, an ACE2 activator, actions on Mas receptor by converting Ang II into Ang 1-7 counterbalances the vasoconstrictive, proliferative, and fibrotic pathways (11). ACE2 is known to improve pulmonary hemodynamics, reduce oxidants and inflammatory mediators, and be well-tolerated in PH patients (5). In line with previous studies (14, 34, 35), our results showed that DIZE has no adverse effect on systemic blood pressure. Moreover, histological examination revealed that DIZE attenuates pulmonary vascular neointimal and medial remodeling in the SuPNx model. We believe this study could be a fundamental study of ACE2 on PH.

In our study, the RVSP in the SUPNx_42_ rats increased significantly, which may favor the assessment of drug effects in preclinical trials. The RVSP improved significantly in both the early and late DIZE treatment protocols, suggesting the effectiveness of hemodynamic changes by DIZE in PAH. However, although the Fulton index did increase in the SuPNx_42_ rats, there were only we can still see the trends of improvement with DIZE in either the early or the late treatment protocols.

In addition to endothelin, RAS has also been implicated as a causative factor in PAH [1]. Ang II, a principal effector peptide of the RAS, can exert deleterious effects on the pulmonary vasculature resulting in vasoconstriction, proliferation, and inflammation, all of which are contributable to the development of PAH. However, it is difficult to measure the plasma and tissue levels of Ang II due to its very short half-life (16 ± 1 s in mice) (36). In contrast, ACE, which catalyzes the conversion of Ang I to Ang II, is abundant in the small pulmonary arteries and is therefore more easily to be detected (37). Thus, measuring ACE, instead of Ang II levels in the lung, provides a more practical method for assessing the associated hemodynamic changes. In our study, there were no significant changes in pulmonary Ang II levels among the four animal groups. However, the expression of pulmonary ACE was increased in SuPNx_42_ rats and ameliorated by DIZE in both early and late treatment group, suggesting that ACE may be a representative marker in this animal model of PAH.

In the RAS, ACE/Ang II/AT1 constitutes the vasopressor arm, which is counterbalanced by the ACE2/Ang-(1-7)/Mas receptor axis (38). By converting Ang II to the vasodilatory peptide Ang-(1-7), ACE2 provides a negative feedback on the RAS and protects the major organs such as heart and kidneys from being damaged by excessive Ang II generated during the development of PAH (39, 40). Interestingly, in the present study, the RV and pulmonary levels of Ang-(1-7) were not significantly altered in the SuPNx_42_ group when compared with the sham-operated rats. However, the RV levels of Ang-(1-7) were significantly elevated in both the early and late DIZE treatment groups, and the pulmonary levels of this peptide were also significantly increased in the early DIZE treatment group.

It should be noted that, besides the action of ACE2, Ang-(1-7) can also be formed by other biochemical pathways. It can be generated from hydrolysis of angiotensin I by neprilysins (NEPs) or cleavage of the Ang-(1-9) by ACE (41–44). With respect to its metabolism, Ang-(1-7) can be subsequently degraded by ACE to form Ang-(1-5), by dipeptidyl peptidase 3 (DPP3) to produce Ang-(3-7) and Ang-(5-7), or by aminopeptidase A (APA) to generate Ang-(2-7) (44). Affecting the activity of any of the aforementioned enzymes will undoubtedly result in a change of the levels of Ang-(1-7). To this end, it is speculated other alternative pathways may also influence the formation of Ang-(1-7) in our animal model. Further studies are needed to fully elucidate the involved biochemical pathways.

It has been reported that Ang-(1-7) can stimulate the releases of endothelial derived nitric oxide (eNOS) and vasodilator prostaglandins as well as potentiate the vasodilatory effect of bradykinin (45–47). Consistent with these results, eNOS was significantly elevated in our early DIZE treatment group when compared to SuPNx_42_ rats (Figure 5B), inferring that Ang-(1-7) may stimulate eNOS release in this animal model. The increased expression of eNOS could contribute to the lowering of pulmonary artery pressure observed herein (Figure 2A).

In summary, our model of hypertensive pulmonary vascular disease in pneumonectomized, SU5416-injected rats resemble the neointimal proliferation and vascular occlusion by smooth muscle cells that occurs in human PAH. The efficacy of DIZE in the early and late treatment group suggests its ability to rescue animals from established hypertensive pulmonary vascular disease. However, the exact mechanisms by which DIZE exerts its beneficial effects remain to be investigated. From our results, it is likely that attenuation of PAH by DIZE involves a combination of antiproliferative effects on pulmonary vascular smooth muscle cells through production of Ang-(1-7), suppression of the growth of vascular smooth muscle cells, and also induction of endothelial cell eNOS expression (47, 48).

There are several limitations of our study. To accurately assess pulmonary hemodynamic parameters in these rats, the selection of a close chest technique would have been more appropriate than an open chest technique and could explain why certain values are abnormally low. In addition, the close chest technique would have allowed access to values of mean pulmonary artery pressure (mPAP) and cardiac output, two other important parameters for evaluating PH severity. Besides, echocardiography is a useful non-invasive screening test for evidence of PH. Therefore, echocardiography would have been useful to validate the fact that SuPNx rats had established PH before the introduction of DIZE treatment in the curative protocol and to follow up its reversal over time.

## Conclusion

The present rat model of PAH showed elevated ACE protein levels in the lung as well as histopathological findings such as Increased intimal and medial fractions accompanied by obliterative lesions. DIZE, an ACE2 activator, reduced RVSP, medial wall thickness, and intimal fraction in pulmonary arterioles. In addition, it upregulates pulmonary expression of the ACE2/Ang-(1-7)/Mas receptor axis, leading to a therapeutic effect in this rat model of PAH. Our study showed that DIZE may be a potential agent for the treatment of PAH.

## Data Availability Statement

The original contributions presented in the study are included in the article/Supplementary Materials, further inquiries can be directed to the corresponding author.

## Ethics Statement

The animal study was reviewed and approved by Kaohsiung Medical University (IACUC approval No. 104226).

## Author Contributions

I-CC, B-NW, and Z-KD: conceptualization. I-CC, C-YC, and Y-CL: methodology. Y-CL, J-YL, Y-CL, and J-HH: validation. I-CC and B-NW: formal analysis. B-NW and Z-KD: investigation. B-NW and Z-KD: resources. I-CC and Z-KD: data curation. Y-CL and I-CC: writing—original draft preparation. B-NW and Z-KD: writing—review and editing. All authors have read and agreed to the published version of the manuscript.

## Conflict of Interest

The authors declare that the research was conducted in the absence of any commercial or financial relationships that could be construed as a potential conflict of interest.

## Supplementary Material

Supplementary Figure 1 The rat model of left pneumonectomy followed by SU5416 injection. Remodeling of neointimal occlusive lesions was observed in this animal model. It has plexiform-like lesions by allowing for the endothelial cell hyperproliferation, a distinct feature shown in human PAH.

Supplementary Figure 2 DIZE did not alter the mean systolic arterial pressure (mSAP). The systolic arterial pressure of each rat was recorded at least in triplicate to average the mSAP. Sham_42_, 123 ± 3 mmHg, *n* = 7; SuPNX_42_, 120 ± 4 mmHg, *n* = 7; SuPNX_42_DIZE_1−42_, 115 ± 4 mmHg, *n* = 6; SuPNX_42_DIZE_29−42_, 114 ± 4 mmHg, *n* = 6 (data represent the mean ± SEM).

Supplementary Figure 3 Relative angiotensin II (Ang II) activity of lung. There is a trend of higher Ang II activity in SuPNX_42_ rats than in matched sham_42_ rats (*p* = 0.0528). There was no difference between SuPNX_42_/DIZE_1−42_ and SuPNX_42_/DIZE_29−42_ rats vs. SuPNX_42_ rats. Values represent the mean ± SEM (*n* = 6–7).

Supplementary Figure 4 The levels of relative ACE2 activity of lung. The level of relative ACE2 activity was significantly lower in the SuPNx_42_ when compared to sham_42_ rats. There is a trend of increased ACE2 activity in SuPNx_42_ /DIZE_1−42_ and SuPNx_42_ /DIZE_29−42_ rats than in SuPNx_42_ rats. Values represent the mean ± SEM. ^*^*P* < 0.05 (*n* = 6–7).
