# Supplementary figures and images for: Angiotensin-Converting Enzyme 2 Activator Ameliorates Severe Pulmonary Hypertension in a Rat Model of Left Pneumonectomy Combined With VEGF Inhibition
Source: Front Med (Lausanne). 2021 Feb 19;8:619133. doi: 10.3389/fmed.2021.619133 (PMC7933511; doi:10.3389/fmed.2021.619133)

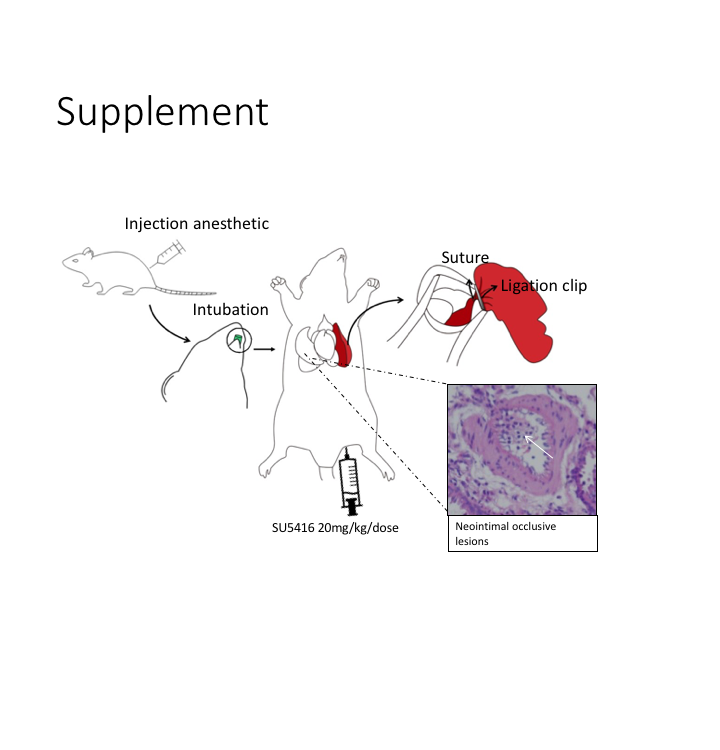

Supplement: Supplementary Figure 1 — The rat model of left pneumonectomy followed by SU5416 injection. Remodeling of neointimal occlusive lesions was observed in this animal model. It has plexiform-like lesions by allowing for the endothelial cell hyperproliferation, a distinct feature shown in human PAH. [file Image_1.TIFF]

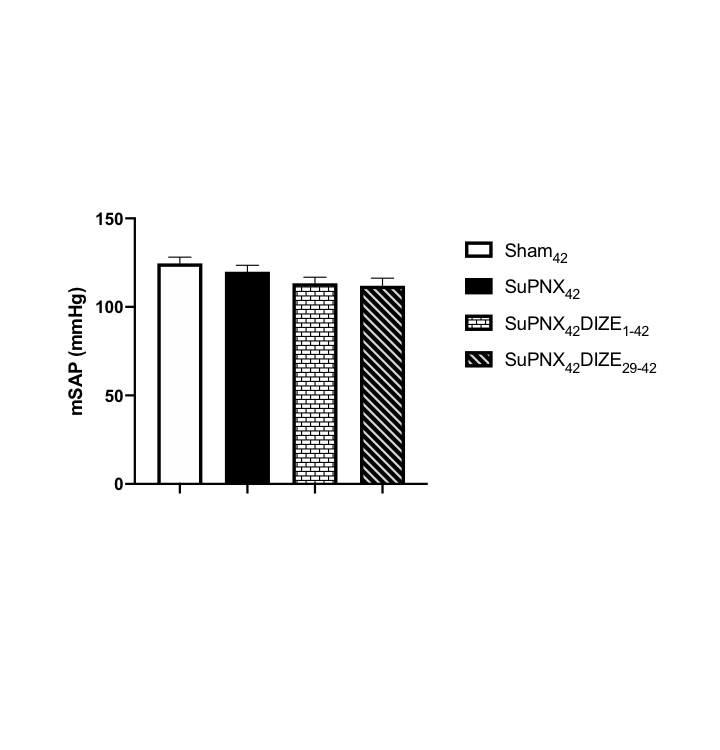

Supplement: Supplementary Figure 2 — DIZE did not alter the mean systolic arterial pressure (mSAP). The systolic arterial pressure of each rat was recorded at least in triplicate to average the mSAP. Sham42, 123 ± 3 mmHg, n = 7; SuPNX42, 120 ± 4 mmHg, n = 7; SuPNX42DIZE1−42, 115 ± 4 mmHg, n = 6; SuPNX42DIZE29−42, 114 ± 4 mmHg, n = 6 (data represent the mean ± SEM). [file Image_2.TIFF]

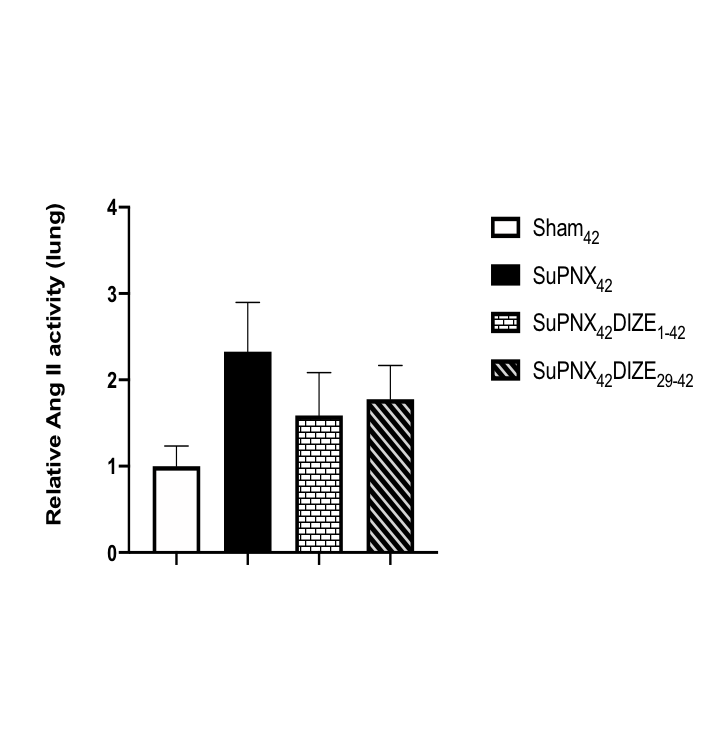

Supplement: Supplementary Figure 3 — Relative angiotensin II (Ang II) activity of lung. There is a trend of higher Ang II activity in SuPNX42 rats than in matched sham42 rats (p = 0.0528). There was no difference between SuPNX42/DIZE1−42 and SuPNX42/DIZE29−42 rats vs. SuPNX42 rats. Values represent the mean ± SEM (n = 6–7). [file Image_3.TIFF]

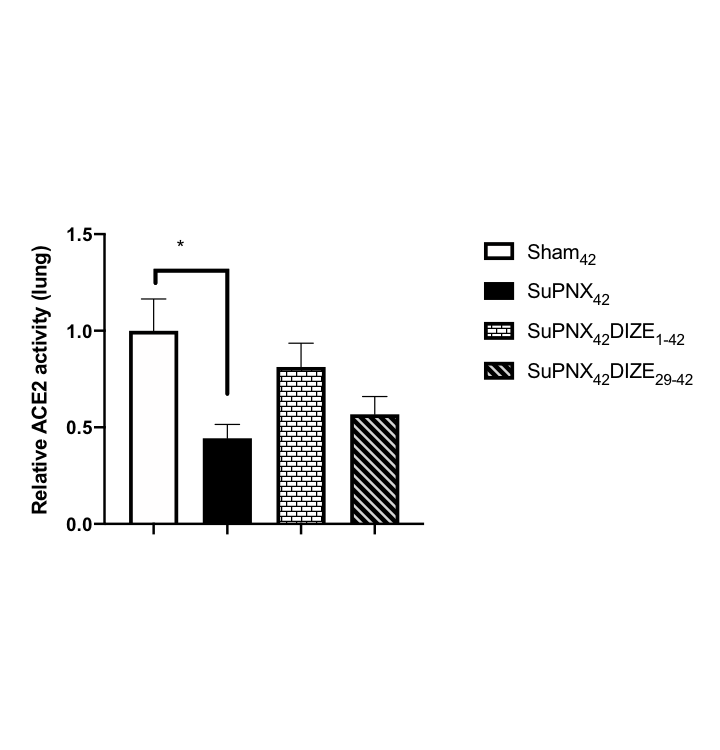

Supplement: Supplementary Figure 4 — The levels of relative ACE2 activity of lung. The level of relative ACE2 activity was significantly lower in the SuPNx42 when compared to sham42 rats. There is a trend of increased ACE2 activity in SuPNx42 /DIZE1−42 and SuPNx42 /DIZE29−42 rats than in SuPNx42 rats. Values represent the mean ± SEM. *P < 0.05 (n = 6–7). [file Image_4.TIFF]
